# iTRAQ-based quantitative proteomic analysis of salt stress in *Spica Prunellae*

**DOI:** 10.1038/s41598-019-46043-9

**Published:** 2019-07-03

**Authors:** Zixiu Liu, Lisi Zou, Cuihua Chen, Hui Zhao, Ying Yan, Chengcheng Wang, Xunhong Liu

**Affiliations:** 1 College of pharmacy, Nanjing University of Chinese Medicine, Nanjing, China; 2 Collaborative Innovation Center of Chinese Medicinal Resources Industrialization, Nanjing, China; 3 National and Local Collaborative Engineering Center of Chinese Medicinal Resources Industrialization and Formulae Innovative Medicine, Nanjing, China; 4 Department of Pharmacy, No. 454 Hospital of PLA, Nanjing, China

**Keywords:** Abiotic, Abiotic, Salt, Salt, Carbon cycle

## Abstract

*Spica Prunellae* is an important Chinese herbal medicine. Because of its good curative effect on various diseases, this herb is consumed in large quantities in clinical applications. The metabolites of *Spica Prunellae* are known to change under salt stress; however, the difference in protein levels of *Spica Prunellae* between saline and normal conditions is unclear. In this study, we used proteomics techniques to identify differentially expressed proteins in *Spica Prunellae* under different saline conditions. (iTRAQ) MS/MS was used to detect statistically significant changes in protein between salt stress and normal conditions. Ultimately, we detected 1,937 proteins, 89 of which were detected in two different comparison. Based on GO, STRING and KEGG analyses, 35 significantly differentially expressed proteins were selected for further analysis. The results of functional and signal pathway analyses indicated that the cellular protein and carbohydrate metabolism of *Spica Prunellae* was weaker, calcium ion transport was higher, photosynthesis was higher, and protein production was faster under saline conditions than under normal conditions. This study provides useful information for studying the causes of differences in secondary metabolites in *Spica Prunellae* under salt stress and the protein mechanisms related to their quality.

## Introduction

Soil salinity is a major external environmental stressor of plants worldwide because it has a very strong negative effect on plant growth, development and production. Under salt stress, aspects of plant growth, including seed germination, root length, and plant height are significantly inhibited^1^. With the rapid development of industry and agriculture, the soluble salt in the soil has increased in recent years. According to reports by the Food and Agriculture Organization (FAO 2008; http://www.fao.org/ag/agl/agll/spush/), more than 400 million hectares of land worldwide have been exposed to high salinity. Irrigation water, which always contains sodium chloride (NaCl), is the main reason for soil salinization worldwide. Soil salinity also affects photosynthesis, protein synthesis, and energy metabolism, and is likely to cause plant physiological drought, ion toxicity, and physiological metabolic disorders^2^. Plants have developed a series of tolerance mechanisms for growth under harmful conditions. This tolerance depends primarily on a variety of biochemical processes that lead to the production of compatible permeants, antioxidants, transporters and specific proteins that control salt damage^3^. For example, plants have developed strategies to reduce osmotic damage, primarily by reducing water loss, while relying on many permeants, organic solutes, inorganic ions, and water channels to maximize moisture absorption^4^. Wiciarz, M revealed the salt tolerance mechanism in *Arabidopsis thaliana* and *Eutrema salsugineum*^5^. StDWF4 overexpression in potato seedlings enables them to resist salt stress by mitigating the negative effects of the stressor^6,7^. Copper, zinc superoxide dismutase (CuZnSOD) and ascorbate peroxidase (APX) overexpression together enhance the tolerance of sweet potato under salt stress^8^. Overexpressing SOD and APX improves salt stress tolerance^9^. Therefore, studying the physiological processes and molecular genetic mechanisms associated with salt stress resistance will certainly help clarify the complex biological responses of plants to high salinity and contribute to the genetic engineering of stress-resistant plants.

*Spica Prunellae* (Xia Ku Cao in Chinese), comprises the fruit spikes of the perennial plant *Prunella vulgaris* L., which is widely distributed in Asia and Europe. As a well-known traditional Chinese medicinal herb, *Spica Prunellae* is commonly used to treat various cancers^10^. *Spica Prunellae* can alleviate liver fire, improve vision, and disperse swelling, and is mainly used to treat eye pain, headaches, dizziness, and swollen breasts^11,12^. Modern clinical practice has demonstrated that the herb possesses anti-inflammatory, antimicrobial, antioxidant, antiviral and immunomodulator properties and can be used to lower blood pressure, alleviate sore throat, reduce fever, treat thyroiditis and accelerate wound healing^13^. *Spica Prunellae* extract reportedly induces programmed apoptosis in human colon carcinoma cell lines^14,15^. *Spica Prunellae* induces efflux transporter expression via activation of the Nrf2-mediated signalling pathway in HepG2 cells^16^. In recent years, as the abundance of *Spica Prunellae* in the wild has declined and the demand for original medicines has risen annually, it has become imperative to carry out work on the artificial cultivation of *Spica Prunellae*. When *Spica Prunellae* is cultivated in the field, many abiotic stress factors affect its yield and quality. Among these stressors, salinity is one of the most severe environmental factors limiting the quality of *Spica Prunellae*. Planting *Spica Prunellae* in salinized land can effectively alleviate the discrepancy between its current supply and strong market demand. Therefore, it is very important to study the salt tolerance mechanism of *Spica Prunellae* to increase the yield and quality of this important herb. There are currently no reports on the mechanism of *Spica Prunellae* response to salt stress.

Proteomics is a frontier method in proteomic research and is widely used in the study of plant resistance to external stress. Proteomics-based technologies have been widely used to identify proteins responsible for salt tolerance in several crops, such as cotton^17^, cucumber^18^ and wheat^19^, promoting advancements in the study of molecular activities under salt stress. iTRAQ technology has been used to identify and quantify 2,165 proteins and 1,815 proteins in developing rice embryos^20^ and wheat grains^19^, respectively.

In this study, a currently popular high-throughput proteomics technology (iTRAQ technology) was adopted to reveal the protein activity of *Spica Prunellae* under salt stress and to study the important protein-mediated stress mechanism of *Spica Prunellae* under salt stress. The energy metabolism and stress-related metabolism of this herb were revealed. The findings of this study provide important reference information and a theoretical basis for future research on salt tolerance in *Spica Prunellae*.

## Material and Methods

### Plant materials and salinity stress

Seedlings of *Spica Prunellae* were collected from Chuzhou City, Anhui Province. The seedlings were sown in plastic pots filled with garden soil and grown under greenhouse conditions (20 ± 2 °C, 50 ± 5% relative humidity, 16/8 h photoperiod) until mature ears were fully developed. For the salt treatments, soluble NaCl was gradually incorporated into the soil until the salt concentrations reached 0 (CK), 50 (Na50), 100 (Na100) or 150 (Na150) mM. The incorporation of NaCl was repeated 5 times for each group. Plants were watered once with Hoagland nutrient solution for ten days. When the plants were approximately five months old (on February 26), the *Spica Prunellae* seedlings were treated with Hoagland nutrient solution as a control or Hoagland nutrient solution with 50, 100 or 150 mM NaCl as a salt stress treatment five times in fifty days. Samples were obtained at 10:00 a.m. on June 14 by pooling the fruit spikes from four plants in the same group, and two replicates were analysed per treatment. Later in sample processing, 30 fruit spikes of *Spica Prunellae* from each group were crushed and mixed in liquid nitrogen, and 5 g of the material from each group was taken as the sample for subsequent proteomic processing.

### Protein extraction

*Spica Prunellae* proteins were extracted using phenol, and few modifications were made^21^. Briefly, the stored *Spica Prunellae* samples were pulverized in liquid nitrogen and then mixed with 10% polyvinylpolypyrrolidone (PVPP). The precipitate was then dissolved in protein extraction buffer containing sucrose (0.8 M), KCl (100 mM), ethylenediaminetetraacetic acid (EDTA) (50 mM, pH of 8.0), phenylmethanesulfonyl fluoride (PMSF) (1 mM), Tris-HCl (50 mM, pH of 8.5), and dithiothreitol (DTT) (1%) at 4 °C. Afterwards, a mixture of equal volumes of Tris-buffered phenol (pH of 8.0) and the sample was thoroughly vortexed. Then, the samples were centrifuged at 12,000 rpm for 30 minutes at 4 °C. The supernatant was carefully removed and back extracted with an equal volume of protein extraction buffer. The mixture was vortexed at room temperature for 5 minutes and centrifuged, and this sequence was repeated 6 times. The final recovered phenol phase was poured into a new tube. Finally, the pellets were air-dried. The protein was then determined using the bicinchoninic acid (BCA) method with bovine serum albumin (BSA) as the standard.

### In-Solution digestion and iTRAQ labelling

After protein quantification, approximately 200 µg of protein was digested with trypsin overnight at 37 °C in a 1:50 trypsin-to-protein mass ratio. After digestion, peptides were reconstituted in 0.5 M triethylammonium bicarbonate (TEAB), and an 8-plex iTRAQ kit (AB Sciex, Framingham, MA, USA) was implemented following the manufacturer’s protocol. Briefly, one unit of thawed iTRAQ reagent was reconstituted in 70 L of isopropanol. Peptides from the salt treatment and control groups were marked with different iTRAQ tags by incubation for 2 h at room temperature. The peptide mixtures were then combined and dried by vacuum centrifugation. The pooled mixtures of labelled peptides were separated by strong cationic exchange (SCX) chromatography.

### hpRP chromatography

High-pH reverse-phase fractionation (hpRP) chromatography was performed using a Dionex UltiMate 3000 high-performance liquid chromatography (HPLC) system with built-in micro collection options for autosampling and ultraviolet (UV) detection. iTRAQ-labelled tryptic peptide was reconstituted in buffer A (20 mM NH_4_HCO_2_, pH of 10) and loaded onto a Gemini-NX C_18_ column (3 μm, 2 × 150 mm, 110 A, Phenomenex) with 20 mM NH_4_HCO_2_ as buffer A and 80% acetonitrile (ACN) + 20% 20 mM NH_4_HCO_2_ as buffer B. The peptide was eluted at a flow rate of 200 μL/min with a gradient of 0–5% buffer B for 10 minutes and then 5–15% buffer B for 5 minutes. Elution with 15–50% buffer B lasted for 45 minutes, and that with 50–90% buffer B lasted for 10 minutes. Twenty-four fractions were collected at 1-minute intervals based on UV absorbance at 214 nm/280 nm and a multi-fraction cascade strategy. Fractions were dried and acidified in 50% trifluoroacetic acid (TFA) for nano-liquid chromatography with tandem mass spectrometry (nano-LC-MS/MS) analysis.

### Nano-LC-MS/MS analysis by a Q exactive

A total of 24 fractions were collected by a linear gradient and acidified with TFA (50%). The fractions were dried in vacuo and further analysed by LC-MS. The fractions (96 μg) were dissolved in buffer A [0.1% formic acid, 2% ACN] and pelleted at 13,500 rpm for 20 minutes. Nano-LC-MS/MS was performed using a Q Exactive mass spetrometer (Thermo Scientific). The supernatant was identified with the Q Exactive system (Thermal Scientific) after being loaded onto analytical columns. Mobile phase A was formic acid (0.1%) and mobile phase B was 80% ACN with 0.1% formic acid. The flow rate was set at 300 nL/min for the analytical columns, and the peptides were analysed with a 3-step gradient (80% ACN in 0.1% formic acid from 4% to 50% over 45 minutes, from 50% to 90% over 5 minutes and kept at 90% for 5 minutes) for 65 minutes. The primary MS parameters included a scan range of 350 to 1,800 m/z with a resolution of 70,000 and a maximum injection time of 40 ms. The second-grade MS spectra were obtained at a resolution of 17,500 with a maximum injection time of 60 ms, and the 20 top precursors per MS cycle were selected.

### Protein identification and data analysis

Protein data analysis was performed according to the method used in a previous study on *Pseudostellaria heterophylla*^22^. Briefly, the initial raw files were converted into mgf files via Proteome Discoverer 1.4 (Thermo, American). Then, Protein Pilot 5.0 (AB Sciex, USA) was used to analyse the mgf files. The Paragon algorithm integrated in Protein Pilot 5.0 was used for database searching. The custom database consisted of protein sequences predicted from RNA data.

The parameter settings were as follows: “Done ID” mode with 95% confidence, iTRAQ peptide labelling, and Cys and trypsin digestion by methyl methanethiosulfonate (MMTS) oxidation. To increase confidence levels, proteins with iTRAQ ratios above 20 or below 0.05 were excluded, and only proteins with a reasonable ratio in all channels were considered quantifiable. Further functional analysis was performed for differential protein expression analysis, including whether the proteins were downregulated or upregulated. The change was determined compared to the CK, and p < 0.05 in the t test was used to indicate a significant difference between *Spica Prunellae* cultivated under salt stress and its blank control. The protein showing an average fold change of ≥ 1.3 or ≤ 0.77 in the experiment and with a minimum of two peptide matches were considered significantly differentially expressed. QuickGO software was used for Gene Ontology (GO) analysis of differentially expressed proteins (DEPs); the software searched the databases most commonly used in bioinformatics research to generate biological process, molecular function and cellular composition information for *Spica Prunellae*. The Kyoto Encyclopedia of Genes and Genomes (KEGG) database (http://www.genome.jp/kegg/pathway.html) was used to exploit the knowledge of current biochemical pathways and other types of molecular interactions.

## Results

### Primary data and protein profiling

**Figure 1:**
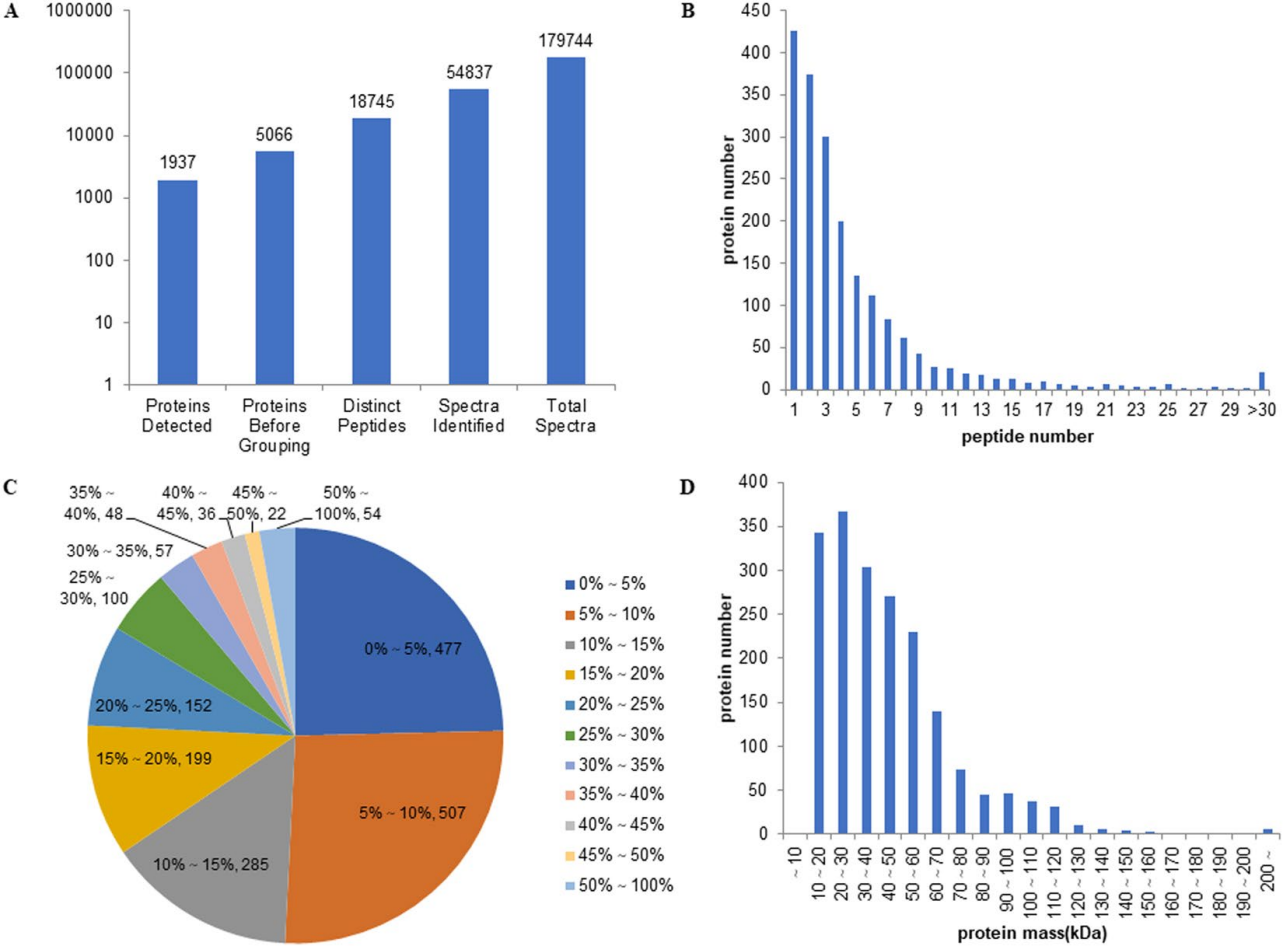
Initial analysis of date and protein identification. (**A**) Basic protein mass distribution. (**B**) Peptide number distribution. (**C**) Protein mass distribution. (**D**) Distribution of proteins sequences coverage.

Mascot software was used to analyse all MS/MS spectra. In total, there were 179,744 spectra, among which 54,837 spectra were quantified. Subsequently, 18,745 distinct peptides were found; based on these peptides, we obtained 5,066 primary proteins, and 1,937 of the primary proteins were retained (Fig. 1A, Table S1). The results of protein sequence and mass analysis indicated that approximately 78% of the identified proteins contained at least two peptides (Fig. 1B). More than 49% of the peptide sequences exhibited a sequence coverage of more than 10%, and more than 24% of the proteins exhibited a coverage of more than 20%, indicating high confidence (Fig. 1C). Regarding the protein mass distribution, 93% of the mass values were between 10 and 100 kDa, indicating a good average coverage.

### DEP classification

**Figure 2:**
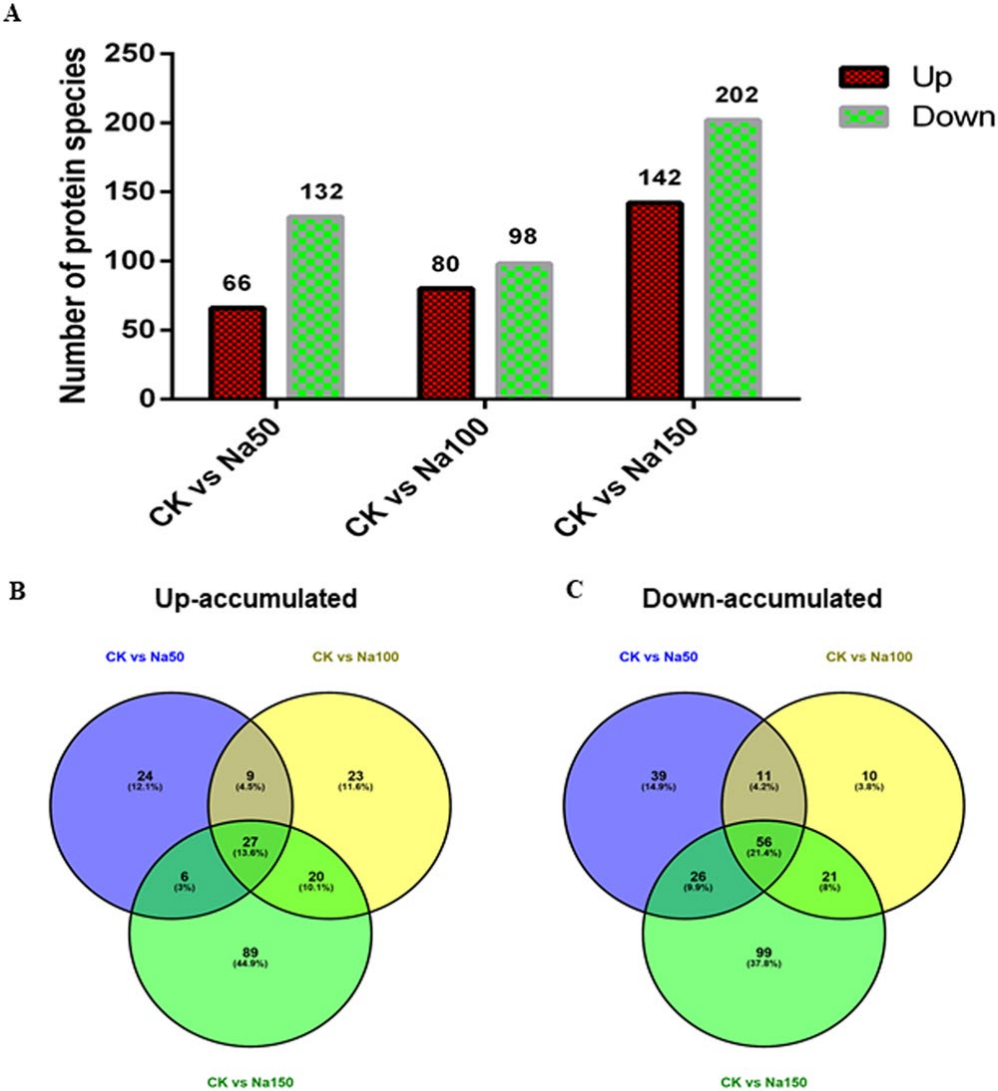
Identification and statistics analysis of DEPs under different salt concentration. (**A**) Number of up- or downregulated proteins between the CK group and different salt concentrations. (**B**) Venn diagram analyses for upregulated proteins. (**C**) Venn diagram analysis for downregulated proteins.

Analysis of changes in protein profiles revealed DEPs (p value ≤ 0.05). Sixty-six proteins in the Na50 group increased in expression by 1.3 times or more and 132 decreased by 0.77 times or less compared to the CK group. The expression level of 80 proteins increased and of 98 proteins decreased in the Na100 group compared with the CK group (178 in total). The level of 142 proteins increased and the level of 202 proteins decreased in the Na150 group compared with the CK group (344 in total) (Fig. 2A, Table S2). Therefore, more protein species displayed a change in abundance in response to the 150 mM NaCl treatment than in response to the other treatments. A Venn diagram of these down- and upregulated protein species between the CK and salt treatments is shown in Fig. 2B,C. We identified proteins that were differentially expressed in *Spica Prunellae* among the three treatments with different salt concentrations. The DEPs of sucrose synthase, pyruvate decarboxylase, bifunctional aspartate aminotransferase, glutamate/aspartate-prephenate aminotransferase, pyruvate, orthophosphate dikinase, D-3-phosphoglycerate dehydrogenase and tyrosine aminotransferase were regulated by only a low salt concentration. These proteins are mainly related to the metabolism of carbohydrates and amino acids, which may indicate that the metabolism of carbohydrates and amino acids in *Spica Prunellae* is abnormally active in the early stage of salt stress to prepare for later adaptation to salt stress. However, the DEPs of glutathione S-transferase, peroxin-14, F-type H+ -transporting ATPase subunit alpha, L-ascorbate peroxidase, nicotinamide adenine dinucleotide (NADH), photosystem II P680 reaction center D1 protein, photosystem II CP47 chlorophyll protein, photosystem II CP43 chlorophyll protein, and photosystem II oxygen-evolving enhancer protein 2 were regulated only after high salt stress. Functional annotations showed that these DEPs were mainly related to photosynthesis and redox functions, which suggested that *Spica Prunellae* was more active in terms of photosynthesis and antioxidant function under high salt stress than under the other concentrations.

### GO and KEGG pathway enrichment analysis for DEPs

**Figure 3:**
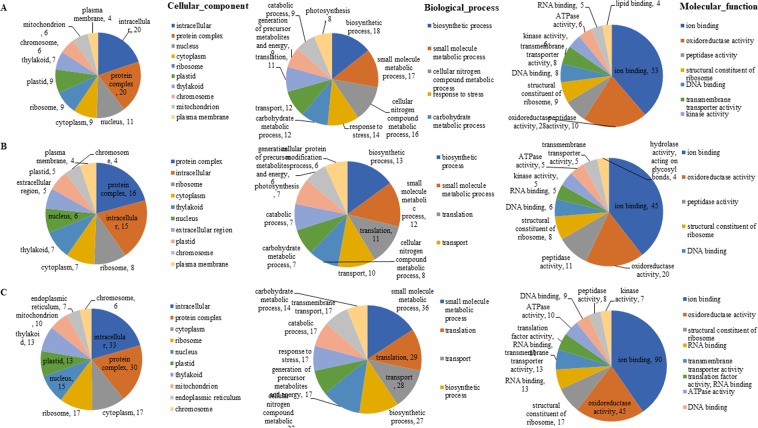
GO assignment of DEPs in cultivated salt stress *Spica Prunellae* and the CK group. (**A**) Differentially expressed proteins in Na50 compare with the CK group. (**B**) Differentially expressed proteins in Na100 compare with the CK group. (**C**) Differentially expressed proteins in Na150 compare with the CK group.

GO and KEGG analysis of these DEPs was performed to unveil the global pattern of protein species abundance. For the GO analysis, comparing the Na50 and CK groups, in the biological process category, 18 proteins were related to biosynthetic process, followed by small molecule metabolic process (17), cellular nitrogen compound metabolic process (16), and response to stress (14). For the cellular component category, 20 proteins were related to intracellular and 20 to protein complex, followed by nucleus (13). In the molecular function category, ion binding was represented by 53 proteins, followed by oxidoreductase activity (28) (Fig. 3A, Table S3). In contrast, in the comparison of the Na100 group with the CK group, in the biological process category, biosynthetic process (with 13 proteins) was the dominant term, followed by small molecule metabolic process (12) and translation process (11). In the cellular component category, a total of 16 and 15 proteins were related to protein complex and intracellular, respectively, while ion binding (45) and oxidoreductase activity (20) were the major functions in the molecular function category (Fig. 3B, Table S4). Furthermore, in the comparison of the Na150 and CK groups, in the biological process category, small molecule metabolic process was detected for 36 proteins, followed by translation (29), transport (28), and biosynthetic process (27). In the cellular component category, the majority of proteins (33) were associated with intracellular, followed by proteins (30), and cytoplasm (17). Finally, in the molecular function category, 90 proteins were related to ion binding, and 45 were related to oxidoreductase activity (Fig. 3C, Table S5).

As shown in Table 1, the 125 DEPs were further investigated using the KEGG database, and in Na50, they were found to be enriched in Carbon metabolism (with 16 proteins), Biosynthesis of amino acids (9), Ribosome (9), Oxidative phosphorylation (8), Photosynthesis (7) and Glycolysis/Gluconeogenesis (7). The top 10 enriched KEGG pathways in the Na100 group are shown in Table 2; Ribosome (with 7 proteins) was the most enriched, followed by Photosynthesis and Carbon metabolism (7), and Biosynthesis of amino acids (6). In the comparison of the Na150 and CK groups (shown in Table 3), Carbon metabolism (with 21 proteins) was the most enriched pathway, followed by Ribosome (20), Biosynthesis of amino acids (14), RNA transport (13) and Photosynthesis (13).

**Table 1:** Top ten KEGG pathways for the identified protein species (CK vs Na50).

KEGG pathway	Pathway Name	Number of proteins
ko01200	Carbon metabolism	16
ko01230	Biosynthesis of amino acids	9
ko03010	Ribosome	9
ko00190	Oxidative phosphorylation	8
ko00195	Photosynthesis	7
ko00010	Glycolysis/Gluconeogenesis	7
ko00710	Carbon fixation in photosynthetic organisms	6
ko00630	Glyoxylate and dicarboxylate metabolism	6
ko00620	Pyruvate metabolism	5
ko04141	Protein processing in endoplasmic reticulum	4

**Table 2:** Top ten KEGG pathways for the identified protein species (CK vs Na100).

KEGG Pathway	Pathway Name	Number of proteins
ko03010	Ribosome	7
ko01200	Carbon metabolism	7
ko00195	Photosynthesis	7
ko01230	Biosynthesis of amino acids	6
ko00010	Glycolysis/Gluconeogenesis	5
ko00190	Oxidative phosphorylation	4
ko00620	Pyruvate metabolism	4
ko04141	Protein processing in endoplasmic reticulum	4
ko04015	Rap1 signaling pathway	3
ko00940	Phenylpropanoid biosynthesis	3

**Table 3:** Top ten KEGG pathways for the identified protein species (CK vs Na150).

KEGG pathway	Pathway Name	Number of proteins
ko01200	Carbon metabolism	21
ko03010	Ribosome	20
ko01230	Biosynthesis of amino acids	14
ko03013	RNA transport	13
ko00195	Photosynthesis	13
ko00190	Oxidative phosphorylation	10
ko00010	Glycolysis/Gluconeogenesis	10
ko04141	Protein processing in endoplasmic reticulum	9
ko00710	Carbon fixation in photosynthetic organisms	8
ko00620	Pyruvate metabolism	7

## Discussion

The ultimate effect of salt stress on plants is protein change. Soil salinity is one of the many serious challenges that plants currently face^23^. Genomics-based technologies, including transcriptomic and proteomic technologies, have been widely used for proteomic analysis in many plant studies^19^. This study represents the first time that iTRAQ has been used to provide a comprehensive explanation and accurate measurement of protein expression at different salt concentrations in the cultivated Chinese traditional herb *Spica Prunellae*. Protein is the ultimate driver of plant life activities. Although the expression level of proteins across the genome is generally stable under normal conditions, it always changes in response to a change in environmental conditions, such as salt stress^24^. Different external environmental factors may induce changes in genome expression, which ultimately results in the differential expression of proteins and leads to the accumulation of differences in secondary metabolic processes^25^. Therefore, proteomics research can help investigate the accumulation of the active constituents of *Spica Prunellae* and the mechanisms that affect its quality. For this research, iTRAQ was used to study the protein changes and related molecular mechanisms in *Spica Prunellae* under salt stress. Based on the results of the GO, KEGG and STRING analyses, 35-significant DEPs were divided into several categories; these DEPs and categories are discussed below (Table 4).

**Table 4:** List of significant differential expressions of protein species identified in *Spica Prunellae* related to salt stress response.

Accession no.	Gene name	Protein name	CK vs Na50a	CK vs Na100b	CK vs Na150c
**Transcription**					
Pr_PRUVU_88145_2	AT2G04520	Nucleic acid-binding, OB-fold-like protein	0.663	0.633	0.575
Pr_PRUVU_11492_1	HTA6	Histone H2A 6	0.707	0.750	0.733
Pr_PRUVU_16589_1	HTA10	Histone H2A 10	0.624	0.699	0.622
**Signal transduction**					
Pr_PRUVU_94782_1	AT1G12310	Calcium-binding EF-hand family protein; Potential calcium sensor	0.617	0.686	0.432
Pr_PRUVU_93904_1	CAM7	Calmodulin 7	0.512	0.709	0.300
Pr_PRUVU_92776_2	CEN1	Centrin2	0.694	0.747	0.484
Pr_PRUVU_46794_1	CAM5	Calmodulin 5	16.955	4.920	6.370
Pr_PRUVU_63581_1	NDPK1	Nucleoside diphosphate kinase 1	0.688	0.730	0.714
Pr_PRUVU_104895_1	ECA3	Calcium-transporting ATPase	2.020	2.809	1.558
**Protein metabolism**					
Pr_PRUVU_83159_1	PBC1	Proteasome beta subunit C1	0.526	0.633	0.476
Pr_PRUVU_102304_1	GDI	RAB GDP-dissociation inhibitor	1.448	1.436	1.330
Pr_PRUVU_109183_1	AT3G05560	60 S ribosomal protein	0.663	0.688	0.440
Pr_PRUVU_22178_1	AT3G05560	60 S ribosomal protein L22-2	0.613	0.666	0.555
Pr_PRUVU_92544_1	AT2G34480	60 S ribosomal protein L18a-2	1.589	1.673	1.799
Pr_PRUVU_31732_1	SKP1	S phase kinase-associated protein 1	0.530	0.740	0.418
Pr_PRUVU_11666_1	AT3G53870	40 S ribosomal protein S3-2	1.426	1.603	2.311
Pr_PRUVU_41109_1	AT1G74060	60 S ribosomal protein L6-2	1.347	1.389	1.327
Pr_PRUVU_40092_1	AT2G09990	40 S ribosomal protein S16 A	1.356	1.381	1.312
**Carbohydrate and energy metabolism**					
Pr_PRUVU_93518_2	ALDH2B4	aldehyde dehydrogenase 2B4	1.489	1.352	2.536
Pr_PRUVU_114134_1	AT2G07698	ATP synthase subunit alpha	1.616	1.690	2.145
Pr_PRUVU_8875_2	ATPA	ATP synthase subunit alpha	0.531	1.387	2.595
Pr_PRUVU_73033_1	CYTC-2	Cytochrome c-2	0.346	0.506	0.172
Pr_PRUVU_7318_1	At1g59900	Pyruvate dehydrogenase complex E1 alpha subunit	1.420	1.427	1.475
Pr_PRUVU_58374_1	AT3G52990	pyruvate kinase	2.463	2.484	3.158
Pr_PRUVU_106457_1	CAD2	Cinnamyl alcohol dehydrogenase 2	2.032	1.746	1.372
Pr_PRUVU_99965_1	GAPC1	Glyceraldehyde-3-phosphate dehydrogenase	1.632	1.461	1.576
Pr_PRUVU_7759_1	GAPB	Glyceraldehyde-3-phosphate dehydrogenase B	1.386	1.933	3.245
Pr_PRUVU_93996_1	AT5G52840	NADH dehydrogenase	0.462	0.647	0.418
**Stress and defense**					
Pr_PRUVU_99871_1	At2g37250	Adenylate kinase	0.726	0.732	0.512
Pr_PRUVU_96062_1	DHAR2	Dehydroascorbate reductase 2	0.720	0.651	0.547
**Photosynthesis**					
Pr_PRUVU_67744_2	PSBD	Photosystem II D2 protein	0.447	1.673	2.221
Pr_PRUVU_58922_1	LHCB4.2	Chlorophyll a-b binding protein CP29.2	0.661	1.456	1.835
Pr_PRUVU_111152_2	CSN6A	COP9 signalosome subunit 6A	0.560	0.598	0.444
**Cell wall and cytoskeleton**					
Pr_PRUVU_89210_1	PRF3	Profilin 3	0.681	0.704	0.626
**Other mechanisms**					
Pr_PRUVU_67323_1	AT3G14540	Terpene cyclase	0.751	2.372	0.552

^a^The abundance ratio of proteins in *Spica Prunellae* under 50 mM NaCl compared to the CK group.

^b^The abundance ratio of proteins in *Spica Prunellae* under 100 mM NaCl compared to the CK group.

^c^The abundance ratio of proteins in *Spica Prunellae* under 150 mM NaCl compared to the CK group.

### Salt stress induces changes in transcription

Nucleic acid-binding, OB-fold-like protein was downregulated in replication, recombination and repair progress. These three functions are very important in RNA transcription^26^. Histone H2A 6 and Histone H2A 10 were downregulated in DNA binding and transcription process; their role is to stabilize the DNA structure and directly increase the transcription of proteins. These proteins participate in the promotion of DNA unwinding, replication and transcription and chromatin structure stability under salinity. This finding is consistent with the findings of previous reports on rice and wheat^27,28^.

### Salt stress induces signal transduction change

Calcium-transporting ATPase and Calmodulin 5 were upregulated while Calcium-binding EF-hand family protein, Calmodulin 7, Centrin2 and Nucleoside diphosphate kinase 1 were downregulated in Signal transduction mechanisms. In plants, the calcium signalling pathway plays a crucial role in initiating complex responses to stress conditions. In our proteomic analysis, several key calcium-signalling components were found to have an altered abundance. It is well established that these DEPs act as important Ca^2+^ signalling sensors in this signalling process^29^. Furthermore, several other protein species related to the calcium signalling pathway, including 14-3-3-like proteins, annexin, calreticulin, phospholipase C (PLC) and phospholipase D (PLD), accumulated under salt stress. For example, 14-3-3 proteins perform a key step in calcium mediated signal transduction by binding to phosphorylated target proteins^30^. Our results suggested that two 14-3-3-like protein species were significantly upregulated under salt stress especially in the Na100 treatment, which was consistent with the results of a previous study on salt-stressed sugar beet^31^. Generally, the abundance of the majority of these signalling-related protein species was strikingly increased by salt stress, implying that the calcium signalling pathway plays an essential and positive role in the *Spica Prunellae* response to high salinity.

### Salt stress induces protein metabolism change

S phase kinase-associated protein 1 was downregulated in ubiquitin-dependent protein catabolic process. Proteasome beta subunit C1 was downregulated in Posttranslational modification, protein turnover, and chaperones, which are included in protein metabolism. There were four upregulated and three downregulated proteins in *Spica Prunellae* that mainly participated in Translation, ribosomal structure and biogenesis in response to salt stress. 60 S ribosomal protein L18a-2, 40 S ribosomal protein S3-2, 60 S ribosomal protein L6-2, and 40 S ribosomal protein S16A were upregulated while 60 S ribosomal protein, 60 S ribosomal protein L22-2, and Nucleic acid-binding, OB-fold-like protein were downregulated in response to salt stress in *Spica Prunellae*. All these proteins participate in protein metabolism. Protein modification, as well as the balance between synthesis and degradation, is a main regulatory pathway that is coordinated to acquire a uniform cellular response to environmental stimulation^32^. Our proteomic analysis showed that 40 ribosomal protein species mainly belonging to two types (40 S and 60 S) exhibited marked overexpression in response to the salt treatments. Moreover, S phase kinase-associated protein 1 was downregulated in ubiquitin-dependent protein catabolic process. Proteasome beta subunit C1 was downregulated in Posttranslational modification, protein turnover, and chaperones, which are included in protein metabolism^33^. Most of these ribosomal protein species were elevated in abundance, potentially suggesting that plants cope with salt stress by accelerating protein synthesis to maintain the balance between the synthesis and degradation of proteins. Nonetheless, an obvious trend was observed in which most of these ribosomal protein species decreased in abundance from the low to high NaCl treatment. A reasonable explanation for this decrease may be that the activity of ribosomes is impaired with an elevated intensity of salt stress. Generally, the differential regulation of distinct translation components suggests that protein biosynthesis might be managed by a complex regulatory mechanism to cope with salt stress in *Spica Prunellae*.

### Salt stress induces carbohydrate and energy transport and metabolic change

Of the proteins that participated in the progression of carbohydrate transport and metabolism related to salt stress, pyruvate kinase, Cinnamyl alcohol dehydrogenase 2, Glyceraldehyde-3-phosphate dehydrogenase and Glyceraldehyde-3-phosphate dehydrogenase B were upregulated under salt stress. Six main DEPs participated in energy production and conversion process; of these DEPs, Aldehyde dehydrogenase 2B4, ATP synthase subunit alpha, ATP synthase subunit alpha, Aldehyde dehydrogenase 2B4, and Pyruvate dehydrogenase complex E1 alpha subunit were upregulated, while Cytochrome c-2 was downregulated. In plants, the glycolysis and tricarboxylic acid (TCA) cycle are principal features of carbohydrate and energy metabolism that not only meet the energy demand but also give rise to many essential cofactors and substrates for other metabolisms^34^. Of these essential cofactors and substrates, the presence of pyruvate kinases suggested the action of glycolysis, which is required by plants for basic metabolism^35^. The abundance of most of these identified glycolytic enzymes was notably upregulated in our proteomic study, possibly indicating that glycolytic activity was induced under salt stress in *Spica Prunellae*. As a downstream reaction for glycolysis products in the mitochondria of aerobic organisms, the TCA cycle is responsible for the oxidation of respiratory substrates to drive ATP synthesis assisted by various enzymes. In the current proteomic study, the abundance of several key enzymes involved in the TCA cycle changed. Similar to the results for glycolysis, these results also indicated that the TCA cycle was active under salt stress, which is in contrast to the results observed for salt-stressed cotton^17^. This discrepancy suggests that a different salt stress mechanism occurs in *Spica Prunellae*. NADPH is involved in the defensive reaction, which can improve resistance to biotic and abiotic stress, such as viral invasion, salt stress, chilling stress, and oxidative stress^36^. Other studies have suggested that a high level of catalase (CAT) activity is necessary for plants to resist oxidative stress; at the same time, CAT is an important scavenger of superoxides^37^. CAT is an important antioxidant enzyme found widely in plants that can enhance defence capabilities^38^, and plays an important role in plant resistance to drought, salinity and heavy metals^39,40^. In conclusion, the number and expression of the upregulated proteins were higher than those of the downregulated proteins, so we surmise that the ability to respond to stress and the catabolism of oxidoreductases in salt-stressed *Spica Prunellae* were slightly stronger than those in the blank group.

### Salt stress induces stress and a change in defences

Adenylate kinase and Dehydroascorbate reductase 2 were downregulated in stress and defense process under salt stress. H_2_O_2_ in roots can be eliminated by the ascorbate-glutathione (AsA–GSH) cycle. In this process, APX reduces H_2_O_2_ to H_2_O using AsA, which undergoes redox reactions catalysed by monodehydroascorbate reductase (MDHAR), dehydroascorbate reductase (DHAR) and glutathione reductase (GR)^41^. The activities and levels of these enzymes exhibited high diversity in roots under salt treatment. Proteomic studies indicate that the expression patterns of Dehydroascorbate reductase 2 vary among plants and salt treatments. Various APX isoforms in NaCl-treated roots of Arabidopsis and rice also showed differential changes between 6 h and 48 h or among different genotypes^42^. These findings reveal that long-term salt treatment affects the AsA-GSH cycle by decreasing the activity of Dehydroascorbate reductase 2 in certain plants. This decrease leads to an increase in the H_2_O_2_ content and damage to plants.

### Salt stress induces photosynthesis protein change

Photosystem II D2 protein and Chlorophyll a-b binding protein CP29.2 were upregulated in Photosynthesis under intermediate and high salt stress but downregulated under low salt stress, while COP9 signalosome subunit 6 A was downregulated, which suggested that salt-tolerant plants increased salt stress by enhancing photosynthesis. This finding is consistent with the previous result. At high salt concentrations, plants enhance energy accumulation by enhancing photosynthesis^40^. Moreover, photosynthesis is a good strategy for resistance to reactive oxygen species (ROS) in plants, and the functional activity of chlorophyll is dependent on a low ROS content. Our results showed that under high salt stress, Chlorophyll a-b binding protein was upregulated to reduce ROS production and enhance photosynthesis to increase material accumulation, thus leading to salt stress resistance, which was consistent with the tolerance mechanism observed in corn^43,44^. Normally, salt stress increases the amount of oxidizing substances in plants. Plants with higher levels of antioxidant ability, either constitutive or induced, reportedly possess greater resistance to different types of environmental stress conditions^23^.

### The regulatory network related to salt stress response

**Figure 4:**
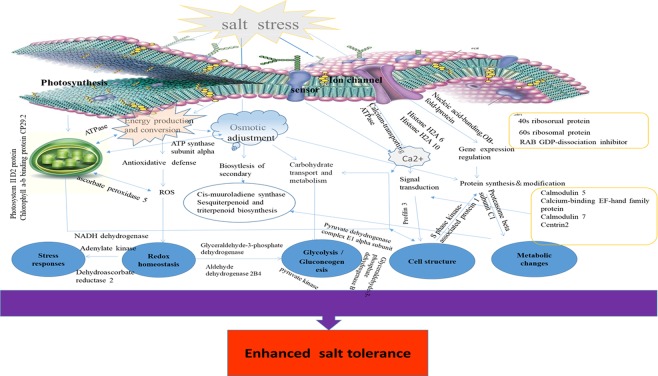
A schematic genetic regulatory network model of salt stress response in *Spica Prunellae*.

The mechanism of salt response in plants is a very complex process in which a variety of genes and response components are involved. In this study, to reveal the molecular mechanism of salt stress response in *Spica Prunellae*, a schematic network model was proposed based on the abundant protein information obtained in conjunction with an association analysis of transcriptomic data (Fig. 4). Stress can cause high concentrations of ROS in plant cells. If these substances are not removed by the antioxidant protection system in time, they will lead to oxidative damage and damage transfer. APX, as well as other peroxidases, such as CAT and SOD, has the ability to scavenge ROS. Salt stress is a kind of environmental adversity. Usually, in adverse environments, oxidative substances such as ROS will accumulate in plants, light-harvesting efficiency will be affected, protein synthesis will slow down, and signal transduction will slow down due to changes in osmotic pressure^45^. In the case of salt stress, plants usually initiate a series of defence mechanisms to transmit signals and activate a series of response mechanisms to increase tolerance. In our study, under salt stress, the accumulation of calcium ions in *Spica Prunellae* increased, and the response of plants to salt stress was activated. At the same time, the signalling molecules of calcium ions were upregulated to help transport calcium ions, which ultimately led to signal transmission and affected the expression of related genes. The gene transcription increased, accompanied by an increase in protein expression and the enhancement of genomic reorganization and repair ability.

Other consequences of salt stress include carbohydrate and protein metabolic disorders, which are all related to the homeostasis and dynamics of cells. Under salt stress, glycolysis and the TCA cycle are activated, resulting in increased energy metabolism. At the same time, protein metabolism is inhibited, more proteins are resistant to salt stress, and photosynthesis is enhanced, leading to more material accumulation, and resistance. Oxidative capacity is enhanced, which indicates that under salt stress, *Spica Prunellae* initiates a series of defence mechanisms to resist salt stress and increase tolerance.

## Conclusions

To study the changes in global proteins under salt stress, we used NaCl as a source of salt stress to perform comparative proteomic analysis of *Spica Prunellae* seedlings. In salt-treated *Spica Prunellae*, 89 protein spots in the Venn diagram were found to be regulated by all three salt concentrations. Of these proteins, 35 were successfully chosen and identified. These identified proteins are involved in different metabolic pathways and processes, including cell rescue/defence, redox homeostasis, signal transduction, photosynthesis, carbohydrate metabolism, nucleotide metabolism, amino acid and nitrogen metabolism, protein biosynthesis, protein folding and assembly, proteolytic proteins, cellular processes, cell wall modifying proteins and unclassified. Some identified proteins may be potential candidates for increasing the salt tolerance of *Spica Prunellae*. Based on the proteomics data, the mechanism of salt stress response involving salt-responsive proteins was further proposed. Such protein analysis will allow us to further understand and describe possible strategies for managing the cellular activities occurring in the ears of salt-treated *Spica Prunellae* seedlings.

## Supplementary information

Dataset 1
